# Items

**Published:** 1909-12

**Authors:** 


### ITEMS.

---

MOET and Chandon White Seal Champagne, is to be preferred for the table, as well as for the sick room. The approach of the holiday season suggests the laying in of good things to eat and drink, and those who enjoy a good sparkling wine,—and who does not,—cannot fail of satisfaction with the Moet and Chandon brand. It is a pure wine, made from choice grapes, and possesses every feature of a high class champagne. For the sick room, too, it is unequaled.

---

APOLLINARIS, the queen of table waters, is an indispensable requisite of the sick room. It is the most palatable of all the sparkling waters, being naturally charged with gas, and is often the most acceptable substance for a sensitive stomach. Mixed with champagne, too, it forms an essential part of the treatment of many conditions otherwise intractable.

